# Rapid vision improvement by using icotinib in a patient with bilateral choroidal metastases symmetrically from lung cancer

**DOI:** 10.1111/crj.13649

**Published:** 2023-06-14

**Authors:** Chaoran Li, Xue E Wang, Lei Yan, Yani Zhao, Yun Kui Zhu

**Affiliations:** ^1^ Department of Respiratory and Critical Care Medicine Xi'an International Medical Center Hospital Xian China

**Keywords:** almonertinib, bilateral choroidal metastases, icotinib, non‐small‐cell lung cancer, positron emission tomography, radiotherapy

## Abstract

**Introduction:**

The metastases of lung cancer to bilateral choroids symmetrically and simultaneously are very rare. Almost all patients with choroid metastasis can be treated with external beam radiotherapy in order to increase quality of life and preserve vision.

**Material and Methods:**

We documented a case and studied the effect of icotinib on choroidal metastases in bilateral eyes simultaneously from pulmonary adenocarcinoma.

**Results:**

A 49‐year‐old Chinese man presented with bilateral vision losing simultaneously for 4 weeks, it was as an initial presentation in the clinical. The examinations with ophthalmofundoscopy, ultrasonography, and fluorescein angiography showed the lesions in bilateral choroids, two solitary juxtapapillary yellow‐white choroidal metastases inferior to the optic discs with bleeding. Positron emission tomography confirmed the choroidal metastases and further proved that it was from lung cancer with lymph nodes and multiple bone metastasis. The biopsy taken from the lung by bronchoscopy and needle biopsy from supraclavicular lymph nodes revealed the pulmonary adenocarcinoma with epithelial growth factor receptor mutation (exon 21). The patient was treated with oral icotinib (125 mg, three times a day, TID). Five days after starting icotinib therapy, the patient's visions were rapidly recovered. Two months after the treatment with icotinib, the choroidal metastases regressed to small lesions, and the visions were preserved to before. The lung tumor and other metastatic lesions were partly regressive. There was no evidence of recurrence for eye lesions at 15‐months follow‐up. After 17 months treating by icotinib, the patient presented headache and dizzy with multiple brain metastases determined by magnetic resonance imaging; however, the lesions of the choroidal metastases remained progressing‐free. Almonertinib with radiotherapy were used to treat the brain metastases, and he is surviving with progress‐free more than 2 years until now.

**Conclusion:**

Bilateral choroidal metastases from lung cancer symmetrically are very rare. Icotinib following by almonertinib was an alternative therapy for choroidal metastasis from non‐small cell lung cancer with epithelial growth factor receptor mutation.

## Graphical abstract

A man presented with vision losing as an initial presentation and ophthalmofundoscopy with PET/CT confirmed the bilateral choroidal metastases from lung cancer. His visions were rapidly improved after 5 days of icotinib treatment with progression‐free survival for up to 17 months. Icotinib following by almonertinib was an alternative therapy for choroidal metastasis of lung cancer.

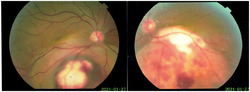

## 1 BACKGROUND

It is well known that lung cancer remains underdiagnosed due to the lack of early‐stage symptoms.^1^ Eye metastasis, including orbit, eyelid, and choroid, is seldom in non‐small cell lung cancer (NSCLC).^2^ Particularly, symmetrical bilateral metastases in choroids presented with both blurred visions as initial symptoms are extremely rare.^3^ Unlike eyelid and orbit, fundus metastasis is easily to be misdiagnosed. Patients with lung cancer who have blurred eyesight, such as blurred vision and eye pain, should be alert to eye metastasis. The ophthalmologist has an important role in diagnosing orbital metastases in lung cancer. Therefore, multidiscipline expertise collaboration is needed to make the diagnosis and determine the prompt treatment in patients.^1^, ^2^, ^3^ Positron emission tomography/computer tomograghy (PET/CT) scan with eye fundus examination is a very important method to reveal the diagnosis, not only of metastatic lesion but also original malignant tumor.^4^ Ocular metastasis treatment is palliative as the presence of metastasis suggests hematogenous spread.^5^ The aim of treatment is to increase quality of life and preserve vision. Choroidal metastasis is rare in the patients with lung cancer, and it may cause visual disturbances that reduce their quality of life. The survival of the patient in choroid metastasis with lung primary is no more than 6 months.^1^, ^2^ External beam radiotherapy are the treatment options available.^2^ Targeted therapy against actionable driver mutations has gradually replaced radiotherapy as the treatment of choice for choroidal metastasis.^6^ It has been reported that erlotinib successfully treated the choroidal metastasis of lung cancer.^7^ The reported case with choroidal metastasis of an anaplastic lymphoma kinase‐rearranged NSCLC received alectinib as the first‐line treatment.^8^ But it has not been reported treating choroidal metastasis from lung cancer with icotinib. We presented a special case and studied the effect of icotinib following by almonertinib on choroidal metastasis in bilateral eyes simultaneously from lung adenocarcinoma.

## 2 CASE PRESENTATION

**FIGURE 1:**
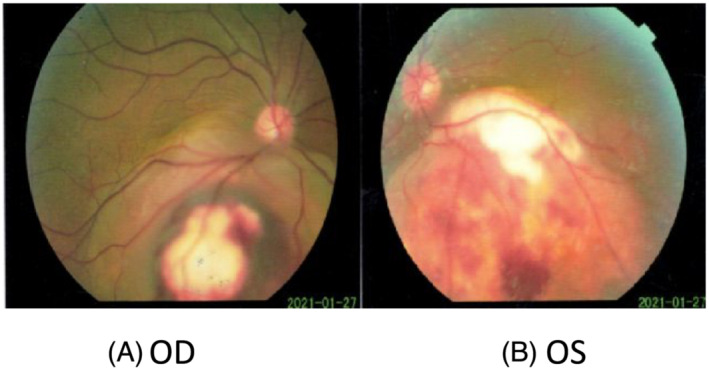
Examination of fundus oculi on January 21, 2021. Ophthalmofundoscopy showed two lesions with the masses under the choroids in right (A, OD, 10 × 7 × 3.2 mm) and left (B, OS,11 × 8 × 2.8 mm) eye grounds, which appeared to be solitary juxtapapillary yellow‐white choroidal metastases inferior to the optic discs with bleeding. This was confirmed by ultrasonography, with subretinal fluid.

**FIGURE 2:**
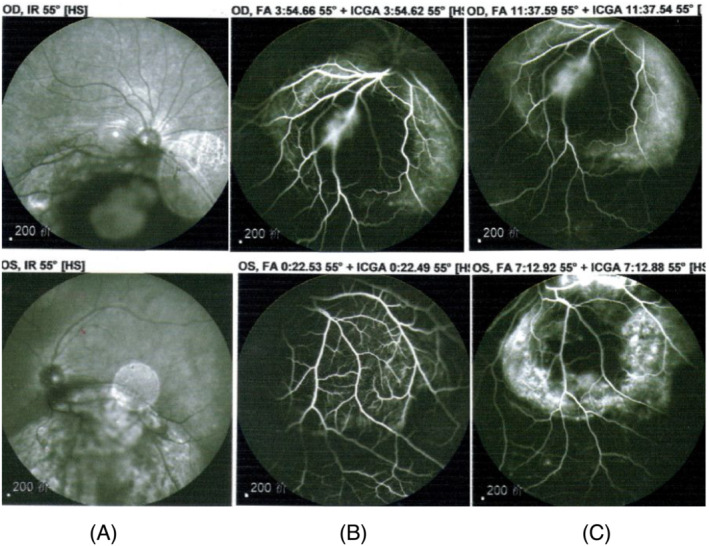
Fluorescein angiography (FA) on January 24, 2021. FA showed the tumor with overlying areas of bright hyperautofluorescence correlating to the deposits of lipofuscin and hyperautofluorescence of subretinal fluid and displayed a hypofluorescent pattern in early arterial phases (A) with hyperfluoresence in the late venous phases (B). Choroidal metastases also contained dilated retinal capillaries with a pinpoint leakage at the tumor border (C).

A 49‐year‐old man was admitted to the Department of Respiratory and Critical Care Medicine of Xi'an International Medical Center Hospital (Shanxi Xi'an, China) in February 2021, because of visual acuity symmetry declined in both eyes for 4 weeks. The patient had no respiratory manifestations including breathlessness, wheezing, cough with bleeding, or expectoration. Decrease in visual acuity was the only initial clinical symptom with daily progress. An ophthalmic examination revealed a reduced visual acuity of 0.10 in the left eye and 0.11 in the right one. In both eyes, the cornea and the anterior chamber were normal and intraocular pressure was 20 mmHg. Ophthalmofundoscopy revealed a yellowish white, elevated choroidal masses in both eye grounds. The masses appeared two solitary juxtapapillary yellow‐white choroidal metastases inferior to the optic discs with bleeding (Figure 1A, 10 × 7 × 3.2 mm in OD[right eye], and Figure 1B, 11 × 8 × 2.8 mm in OS [left eye]). It was confirmed by ultrasonography, with subretinal fluid. Fluorescein angiography (Figure 2A–C) showed the tumor with overlying areas of bright hyperautofluorescence correlating to the deposits of lipofuscin and hyperautofluorescence of subretinal fluid and displayed a hypofluorescent pattern in early arterial phases with hyperfluoresence in the late venous phases. Choroidal metastases also contained dilated retinal capillaries with a pinpoint leakage at the tumor border (Figure 2C).

**FIGURE 3:**
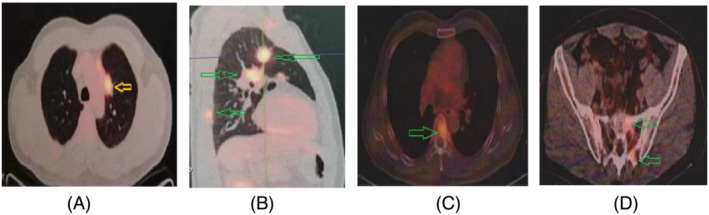
Positron Emission Tomography/CT scan on January 28, 2021. Positron Emission Tomography/CT scan showed a hypermetabolic focus in left lung (SUVmax = 8.7, A) with multiple lymphatic metastases of hilar and mediastinum (B) and multiple bone metastases (B–D, ribs, vertebra, and pelvic bones). Positron Emission Tomography/CT revealed the lung cancer with systemic metastases.

**FIGURE 4:**
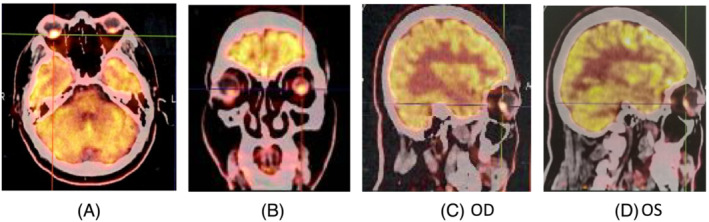
Positron Emission Tomography/CT scan on January 28, 2021. It showed the hotspots increasing fluorodeoxyglucose (FDG) uptake in the regions of the right (SUVmax = 4.8) and left (SUVmax = 5.6) eye funds, suggestive of a metastasis of malignant in both choroids symmetrically from lung cancer.

Thoracic CT scan on February 27, 2021, showed a nodule, 17 × 21 mm in diameter, with burrs and enlarged mediastinal lymph nodes. PET/CT scan determined the nodule with hypermetabolic lesions (SUVmax = 8.6; Figure 3A) in left upper lung with the metastases of hilar and mediastinal lymph nodes (Figure 3B) and metastatic lesions of multiple bones (rib, vertebra, and pelvic bone; Figure 3C,D). Further, PET/CT scan showed metastatic malignant tumors with higher hypermetabolic lesions (SUV = 4.8 in OD and SUV = 5.6 in OS) in both choroids symmetrically (Figure 4A–D) without brain metastasis. PET/CT examination was completely consistent with fundus and FA examinations.

**FIGURE 5:**
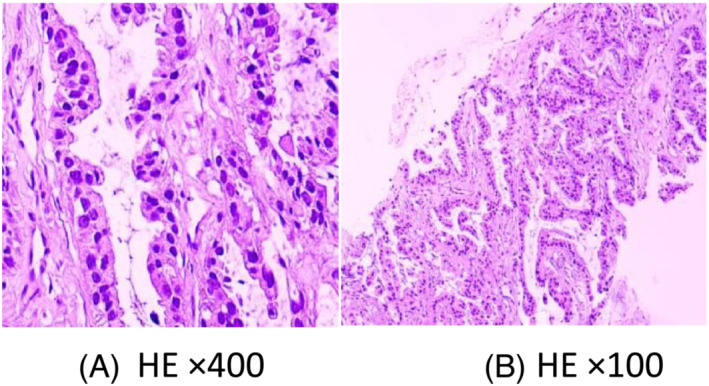
Pathological diagnosis on January 30, 2021. Pathological diagnosis confirmed lung adenocarcinoma (A) with supraclavicular lymphatic metastases (B).

**FIGURE 6:**
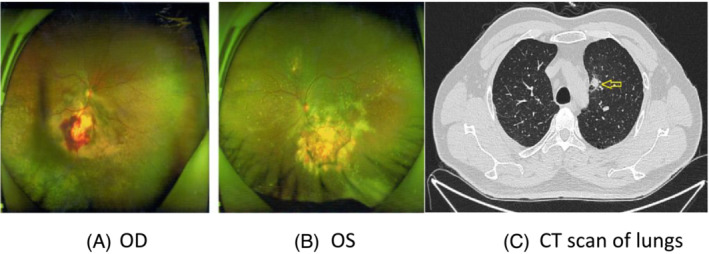
Eye fundus examination and CT scan of lung in March 28, 2021. Ophthalmofundoscopy showed that bilateral choroidal lesions were significantly improved with (A, B) a little bleeding and exudation in the choroids. The CT scan of the lung showed that the nodule in left upper lung significantly shrank (C).

**FIGURE 7:**
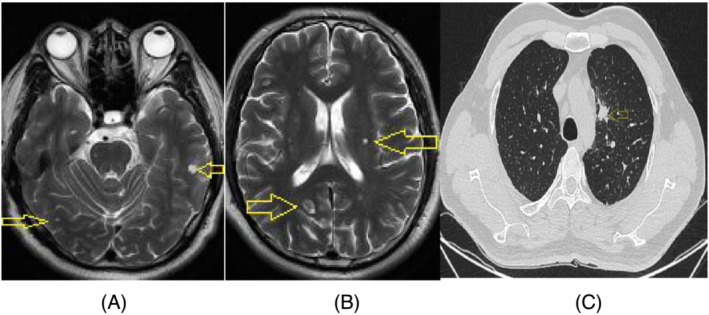
Magnetic Resonance Imaging (MRI) of brain and CT scan of lung on July 20, 2022. Seventeen months after Icotinib treatment, MRI of brain showed that multiple metastases (14 lesions) appeared in brain. But the bilateral choroidal lesions could not be determined by MRI. The CT scan of the lung showed the nodule in left upper lung did not significantly progress (C).

**FIGURE 8:**
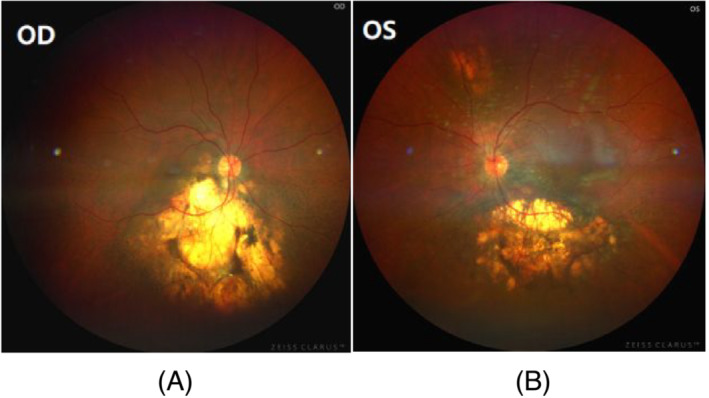
Examination of ophthalmofundoscopy on July 20, 2022. Seventeen months after Icotinib treatment, ophthalmofundoscopy showed that metastatic lesions did not significantly grow in bilateral choroids in (A) OD and (B) OS, with atrophic and cicatricial lesions.

Examination of two biopsies taken from the left upper lung by bronchoscopy and the supraclavicular lymph node by fine needle aspiration revealed pulmonary adenocarcinoma (Figure 5A,B). Icotinib (first generation tyrosine kinase inhibitor [TKI]) was administrated in a dose of 125 mg (three times a day, [TID]) without adequate information of epithelial growth factor receptor (EGFR) because of refusing chemotherapy by patient. However, the visions quickly regained 5 days after using icotinib. After 7 days, EGFR mutation was detected by next generation sequence (NGS) that revealed EGFR exon 21 (L858) with a positive mutation. Owing to systemic and suspicious intracranial metastasis of pulmonary adenocarcinoma, osimertinib was administrated in a dose of 80 mg/daily to replace icotinib in order to get more effective treatment. However, an acute severe liver injury occurred in the patient after 10 days, with high alanine aminotransferase (ALT) and asparate aminotransferase (AST) leves, as well as jaundice. Osimertinib had to be replaced by icotinib. Two months later, the visions improved continually, and the lesions of choroids were significantly improved by the fundus examination (Figure 6A,B). The chest CT scan showed the tumor in the lung, and the lymphatic metastases in the mediastinum were in partial regression (Figure 6C). Icotinib was administered persistently; until in July 2022, the patient presented with headache and dizzy. Magnetic resonance image (MRI) revealed multiple brain metastases (Figure 7A,B). On contrary, the fundus examination and MRI revealed that the choroid metastatic lesions did not significantly progress with atrophic, cicatricial lesions (Figure 8A,B). T790 M mutation of EGFR was positive by second NGS test in the blood. Almonertinib (third generation TKI) was administered at a dose of 110 mg/per day, and whole brain radiation therapy was completed (20 Gy). After 3 weeks, his dizzy and headache were gradually improved. The performance status of the patient was evaluated as 1. The visual acuity of bilateral eyes was remained in premorbidity status. The patient is surviving with normal visions more than 2 years until now by following up.

## 3 DISCUSSION

Malignant tumors from distant organs of the body can spread to the eye. Breast is the most common site for choroid metastasis followed by lungs.^1^ The incidence of metastasis of lung carcinomas into choroid is 2–6.7%,^1^, ^2^ and it is significantly less than brain metastasis. Though choroid metastasis as initial presentation is rare,^2^ patient presented with an ocular tumor should undergo a thorough systemic examination. 20% to 40% of choroidal metastases are bilateral.^1^ But the symmetric and simultaneously metastasized into choroids without brain metastasis is extremely rare in lung cancer. Choroidal metastasis diagnosed primarily on clinical findings needs supplementation of imaging studies. Ultrasonography, fluorescein angiography, optical coherence tomography, MRI, and fine needle aspiration cytodiagnosis are various modalities for diagnosis.^1^, ^2^ In our case, PET/CT scan had been performed early, and it suggested that PET/CT could explore not only the choroidal metastases but also primary site of the tumor and further providing an approach for biopsy. With eye fundus examinations, PET/CT is a very important method to reveal the diagnosis of choroidal metastasis and original malignant tumor. Eye metastasis diagnosed primarily on clinical findings needs supplementation of imaging studies including MRI and PET/CT.^4^ Biopsy taken from lung by bronchoscopy and supraclavicular lymph nodes by fine needle aspiration is much easy than fine needle aspiration cytodiagnosis through eye.

Ocular metastasis treatment is palliative as the presence of metastasis suggests hematogenous spread.^5^ The aim of treatment is to increase quality of life and preserve vision.^3^, ^5^ The survival of the patient in choroid metastasis with lung primary is no more than 6 months.^1^, ^2^ Choroid metastasis not destroying the center of the retina offers the best hope for improving the vision. External beam radiotherapy, plaque radiotherapy, surgical resection, transpupillary thermotherapy, and intravitreal chemotherapy are the treatment options available.^9^ However, the targeted therapy against actionable driver mutations has gradually replaced radiotherapy as the treatment of choice for choroidal metastasis.^6^, ^10^ Novel therapies based on molecular pathways are now commonly used in the treatment of lung cancer as first line for NSCLC.^6^, ^7^, ^8^, ^9^, ^10^ TKIs are effective against eye metastasis if mutations were detected by NGS. Both the primary tumor type and local tumor invasion are the major factors determining the survival for choroidal metastasis. The survival of choroid metastasis with lung primary ranges from 0.5 to 19 months.^1^, ^2^ Mean survival time following diagnosis of ocular metastases was 12 months.^2^ A case reported showed a complete regression of choroid metastasis 2 weeks after starting treatment with oral erlotinib and with persistent regression at 5 months follow‐up.^7^ Successful treatment with alectinib for choroidal metastasis in anaplastic lymphoma kinase rearranged NSCLC.^8^ Generally, the patient with choroidal metastasis could be considered as with brain metastasis, and the third generational TKI should been used in the first line if EGFR mutation is positive, such as osimertinib. An acute liver injury with high ALT and AST as well as jaundice occurred in our patient after osimertinib treatment; it had to be ceased after 10 days. Icotinib was reused to observe the effects on liver function; finally, it was safe to our patient. Icotinib was administered persistently until the brain metastases occurred. However, even if the brain metastasis was revealed, the choroidal metastases in the patient was still without progression. After icotinib, another third TKI, almonertinib, was administered with whole brain radiotherapy. The patient is surviving progressive‐free more than 2 years by following up to now. Thus, the case highlights that presentation with blurred visions as initial symptoms, physician should consider the possibility of metastasis from lung cancer, and with eye fundus examinations, PET/CT is a very important method to reveal the diagnosis of choroidal metastasis and original malignant tumor. Targeting therapy with TKI is crucial treatment for the patient with EGFR mutation.

## 4 CONCLUSION

Bilateral choroidal metastases from lung cancer symmetrically are rarely seen, and icotinib following by almonertinib was an alternative therapy for choroidal metastasis from NSCLC with EGFR mutation.

## AUTHOR CONTRIBUTIONS

ChaoRan Li and Yunkui Zhu finished study design and manuscript editing as well as patient's diagnosis and treatment. XueE Wang, LeiYan, Yani Zhao finished study and patient's treatment and following up in this year. All authors read and approved the final manuscript.

## CONFLICT OF INTEREST STATEMENT

We declare no competing interests. We declare no any disclosure about the patient in the manuscript.

## ETHICS STATEMENT

The research was approved by the Ethics Committee of Xi'an International Medical Center Hospital, and all participants provided informed consent.

## AUTHOR INFORMATION

Chaoran Li, the first author, Department of Respiratory and Critical Care Medicine of Xi'an International Medical Center Hospital.

Email: lcrbing@126.com; telephone number: 0086‐029‐68302596.

XueE Wang, the second author, Department of Respiratory and Critical Care Medicine of Xi'an International Medical Center Hospital.

Email: wangxue202205@163.com; telephone number: 0086‐029‐68302596.

LeiYan, the third author, Department of Respiratory and Critical Care Medicine of Xi'an International Medical Center Hospital.

Email: yanlchn@163.com; telephone number: 0086‐029‐68302596.

Yani Zhao, the fourth author, Department of Respiratory and Critical Care Medicine of Xi'an International Medical Center Hospital.

Email: lpfchd_2008@163.com; telephone number: 0086‐029‐68302596.

YunKui Zhu*, corresponding author, Department of Respiratory and Critical Care Medicine of Xi'an International Medical Center Hospital.

Email: yunkuizhu@qq.com; telephone number: 0086‐029‐68302598.

## Data Availability

Research data are not shared.
